# Omics in Clinical Practice: How Far Are We?

**DOI:** 10.3390/diagnostics12071692

**Published:** 2022-07-11

**Authors:** María Eugenia Sáez

**Affiliations:** Andalusian Bioinformatics Research Centre (CAEBi), 41013 Sevilla, Spain; mesaez@caebi.es

## Summary

The recent development of high-throughput omics technologies has revolutionized the fields of molecular diagnosis and drug development, providing detailed information of cell biology at a degree of resolution never seen before. This huge amount of information requires specialized methods, which are, in fact, continuously growing and diversifying.

In this Special Issue, we focus on the use of multi-omics data for the diagnosis of common complex diseases. These diseases do not follow the Mendelian inheritance patterns, and harbouring a predisposing allele does not guarantee the appearance of the disease since. Other genetic factors, life habits and environmental factors have also a prominent role in the disease to complicate things further [1].

The report from Madrid et al. [2] focusses on the identification of plasma biomarkers of Alzheimer’s disease (AD), the leading cause of dementia worldwide. Although the presence of amyloid deposits and neurofibrillary tangles in the brain are hallmarks of the disease, the identification of peripheric markers of the pathology has turned out a complicated task. In fact, only amyloid and tau levels in cerebrospinal fluid (CSF) are currently used in the clinical setting. Madrid et al. identified genes whose expression was altered in the presence of polymorphic variants previously associated with AD by means of eQTL (expression Quantitative Trait Locus) analysis and compared their expression levels on blood and brain of AD cases and cognitive normal controls for identifying candidate biomarkers of AD.

Corma-Gómez et al. [3] performed a genome-wide association analysis for unrevealing genes that could be involved in the aetiopathogenesis of liver stiffness after antiviral therapy for hepatitis C infection. Subsequent system biology approaches revealed conformational changes in DNA as a key element of the process, allowing the early diagnosis of subjects at risk of developing this unwanted side effect of hepatitis therapy.

Although most commonly used biomarkers are blood-based, CSF, urine, saliva or breath are other frequent sources of biological material for diagnostics purposes. In this issue, Dima et al. [4] performed a systematic review of the use of volatile organic compounds (VOCs) in exhaled breath as non-invasive biomarkers in digestive neoplasia.

Finally, two reports from Siristatidis et al. [5,6] explore the use of omic data and artificial intelligence solutions for improving the success of in vitro fertilization (IVF) with a focus on metabolomic profiling, an application of enormous clinical and economic importance.
